# Supplementary material for: Isolation and Functional Characterization of MsFTa, a FLOWERING LOCUS T Homolog from Alfalfa (Medicago sativa)
Source: Int J Mol Sci. 2019 Apr 22;20(8):1968. doi: 10.3390/ijms20081968 (PMC6514984; doi:10.3390/ijms20081968)
Supplement: Supplementary file 1 [file ijms-20-01968-s001.pdf]

Table S1. Homology matrix of some FT-like proteins in rice, soybean, barley, barrel clover, poplar and alfalfa.

Gene																										AtFT	Mt6G03	MtFTc	MtFTb2	MtFTb1	MtFTa2	MtFTa1	MsFTa
Hv1Hv1g076430	1.00																																
Hv2Hv1g023180	0.59	1.00																															
hv2Hr1g084540	0.51	0.54	1.00																														
Hv3Hr1g027590	0.65	0.62	0.59	1.00																													
Hv3Hr1g087100	0.63	0.62	0.63	0.80	1.00																												
Hv4Hr1g012200	0.58	0.54	0.76	0.64	0.69	1.00																											
Hv7Hr1g024610	0.63	0.62	0.63	0.80	1.00	0.69	1.00																										
Pt010g179700	0.63	0.61	0.67	0.78	0.82	0.69	0.82	1.00																									
Pt008g077700	0.64	0.63	0.67	0.80	0.83	0.70	0.83	0.91	1.00																								
Gm18g298900	0.55	0.54	0.56	0.64	0.62	0.61	0.62	0.71	0.69	1.00																							
Gm18g299000	0.55	0.54	0.61	0.67	0.70	0.63	0.70	0.72	0.73	0.76	1.00																						
Gm16g151000	0.63	0.61	0.61	0.75	0.77	0.64	0.77	0.82	0.83	0.67	0.67	1.00																					
Gm16g044200	0.56	0.58	0.63	0.69	0.74	0.67	0.74	0.79	0.79	0.65	0.67	0.78	1.00																				
Gm19g108100	0.57	0.58	0.63	0.70	0.74	0.67	0.74	0.78	0.80	0.65	0.67	0.78	0.99	1.00																			
Gm16g150700	0.64	0.61	0.60	0.74	0.78	0.65	0.78	0.82	0.83	0.67	0.67	0.96	0.78	0.77	1.00																		
Gm16g044100	0.61	0.61	0.56	0.66	0.67	0.61	0.67	0.72	0.70	0.66	0.61	0.68	0.62	0.62	0.69	1.00																	
Gm19g108200	0.59	0.58	0.56	0.65	0.65	0.62	0.65	0.70	0.69	0.64	0.61	0.67	0.61	0.61	0.68	0.96	1.00																
Gm08g363100	0.57	0.55	0.59	0.64	0.64	0.59	0.64	0.70	0.72	0.68	0.67	0.70	0.65	0.65	0.69	0.61	0.57	1.00															
Os04g0488400	0.53	0.55	0.89	0.62	0.67	0.80	0.67	0.70	0.70	0.60	0.64	0.66	0.68	0.68	0.65	0.60	0.60	0.62	1.00														
Os05g0518000	0.72	0.57	0.53	0.62	0.63	0.56	0.63	0.64	0.66	0.53	0.53	0.61	0.57	0.57	0.62	0.61	0.58	0.57	0.53	1.00													
Os06g0157500	0.61	0.60	0.60	0.75	0.86	0.62	0.86	0.77	0.75	0.60	0.66	0.72	0.69	0.69	0.72	0.65	0.64	0.61	0.61	0.59	1.00												
Os06g0552900	0.61	0.87	0.54	0.62	0.62	0.57	0.62	0.62	0.64	0.56	0.55	0.62	0.59	0.59	0.62	0.61	0.58	0.56	0.56	0.57	0.61	1.00											
Os11g0293800	0.57	0.56	0.79	0.69	0.72	0.87	0.72	0.75	0.75	0.65	0.67	0.69	0.73	0.73	0.68	0.61	0.61	0.65	0.82	0.57	0.67	0.58	1.00										
Os12g0232300	0.54	0.53	0.68	0.64	0.62	0.72	0.62	0.65	0.67	0.53	0.57	0.62	0.62	0.62	0.62	0.57	0.58	0.56	0.72	0.53	0.58	0.54	0.74	1.00									
Os06g0157700	0.61	0.62	0.64	0.80	0.91	0.66	0.91	0.81	0.82	0.63	0.69	0.77	0.74	0.73	0.78	0.66	0.65	0.64	0.67	0.62	0.86	0.63	0.72	0.63	1.00								
AtFT	0.58	0.62	0.65	0.71	0.72	0.68	0.72	0.78	0.79	0.62	0.63	0.73	0.74	0.74	0.73	0.66	0.66	0.64	0.70	0.59	0.69	0.64	0.71	0.64	0.73	1.00							
Mt6g033040	0.60	0.61	0.61	0.70	0.73	0.65	0.73	0.80	0.79	0.67	0.69	0.86	0.78	0.77	0.86	0.70	0.68	0.67	0.65	0.61	0.71	0.61	0.69	0.62	0.74	0.71	1.00						
MtFTc	0.54	0.54	0.54	0.61	0.62	0.59	0.62	0.68	0.67	0.59	0.57	0.64	0.60	0.60	0.66	0.76	0.76	0.57	0.57	0.56	0.64	0.55	0.57	0.51	0.61	0.61	0.64	1.00					
MtFTb2	0.60	0.54	0.59	0.68	0.69	0.64	0.69	0.74	0.74	0.70	0.70	0.69	0.72	0.71	0.70	0.65	0.63	0.74	0.62	0.57	0.65	0.54	0.68	0.59	0.69	0.65	0.71	0.60	1.00				
MtFTb1	0.61	0.55	0.60	0.68	0.69	0.66	0.69	0.75	0.74	0.69	0.70	0.70	0.71	0.70	0.70	0.67	0.65	0.72	0.64	0.59	0.68	0.57	0.69	0.60	0.69	0.69	0.72	0.63	0.92	1.00			
MtFTa2	0.57	0.55	0.61	0.67	0.70	0.62	0.70	0.70	0.74	0.56	0.59	0.70	0.77	0.77	0.70	0.60	0.57	0.59	0.64	0.59	0.64	0.54	0.65	0.59	0.69	0.67	0.65	0.55	0.65	0.65	1.00		
MtFTa1	0.57	0.54	0.63	0.70	0.73	0.65	0.73	0.77	0.77	0.59	0.63	0.75	0.83	0.83	0.75	0.61	0.59	0.65	0.67	0.57	0.69	0.57	0.69	0.61	0.72	0.73	0.73	0.60	0.69	0.72	0.77	1.00	
MsFTa	0.57	0.54	0.64	0.70	0.72	0.66	0.72	0.76	0.76	0.60	0.64	0.74	0.84	0.84	0.74	0.61	0.59	0.65	0.67	0.57	0.68	0.56	0.70	0.60	0.73	0.72	0.74	0.59	0.70	0.72	0.77	0.99	1.00

Homology matrix of FT-like proteins in the indicated species. Note: the bottom row listed the sequence homology of MsFTa with 32 FTs from the indicated plant species. The identity with MtFTs was shaded, and the identity of Mt6g033040 with its paralogs was boxed. The lowest and highest identities with FTs from non-medicago species were underlined.

Table S2. Primers used in this study.

Name	Sequence	Note
5'-RACE	5'-ATCGTTTCCACCAACACTCACTCTGGG-3'	
3'-RACE	5'-ATGGCTGGTAGCAGTAGGAATCCACTGG-3'	
degenerate primer	5'-ATG(A/C/G)TGGTAG(T/C)AG(T/A)(A/C)(G/C)AA(T/C)CC-3'	
degenerate primer	5'- T(C/T)ATCTAAA(C/G)(C/G)T(T/C)CTTCCNCCAGA-3'	
pBI-MsFTa -f	[1]5'-CCCGGGATGGCTGGTAGCAGTAGGAATCCA-3′	Construction of a 35S::*MsFTa* ORF binary vector using XbaI-BamHI for *Arabidopsis* transformation; also used in verification of transgenic plants.
pBI-MsFTa -r	[2]5'- TCTAGATTATCTAAAGGTTCTTCC-3′	
FT-GF	[3]5'-CTCGAGATGGCTGGTAGCAGTAGGAATCCAC-3′	Sub-cloning of *MsFTa* into a GFP fusion binary vector for transient expression
FT-GR	[4]5'-ACTAGTTCTAAAGGTTCTTCCGCCAGAGCCAC-3′	
MsFTa - 1	5'-TGTATCACCATGAGTCAAGACATTG-3'	*MsFTa*-specific primers for RT-PCR
MsFTa -2	5'-CAATGTCTTGACTCATGGTGATACA-3'	
MsFTa1G-f	5'-ATGGCTGGTAGCAGTAGGAATCCACT-3'	Amplification of genomic sequence of MsFTa
MsFTa1G-r	5'-TTATCTAAAGGTTCTTCCTCCAGAGCCACTC-3'	
MsActin-1	5'-CAAAAGATGGCAGATGCTGAGGAT-3'	RT-PCR of *Actin* from alfalfa

MsActin-2	5’-CATGACACCAGTATGACGAGGTCG-3’	
35S promoter-f	5’- CACTATCCTTCGCAAGACCC-3’	Verification of transgenic Arabidopsis using genomic DNA
MsFT-3r	5’-TCTAAAGGTTCTTCCGCCAGAGCC-3’	
Actin-1	5' -GAAGTCTTGTTCCAGCCCTCGTTTG -3'	RT-PCR of *Actin2* (*At3g1878*0) from Arabidopsis
Actin-2	5' –GAACCACCGATCCAGACACTGTACT-3’	

Underlined sequences are recognition sites for [1]XbaI, [2]*Sma* I, [3]*Xho* I and [4]*Spe* I, respectively.

International Journal of
Molecular Sciences

MDPI

```
TAAATTCAAGCAGAAAGATTGGTACGCAACAGGAAAGGAATATTTATCCATGACCTCTATCTTTCTTCT
ATAAATACCTCTAGCAATTGGTGGTTACCCTAAGACAAATTTGTGTATAAAAGTGTGAGAACTGAGAAC
CACATATGGCTGGTAGCAGTAGGAATCCACTGGCTGTAGGGCGTGTAATAGGGGATGTGATAGACTCCT
    M   A   G   S   S   R   N   P   L   A   V   G   R   V   I   G   D   V   I   D   S
TTGAAAGTTCCATTCCTCTCCGAGTGACCTATGGTAATAAAGATGTGAATAATGGTTGTGAGCTCAAAC
F   E   S   S   I   P   L   R   V   T   Y   G   N   K   D   V   N   N   G   C   E   L   K
CTTCTCAAATTGGCAATCAACCCAGAGTGAGTGTTGGTGGAAACGATCTCAGAAACCTCTACACCCTAG
P   S   Q   I   G   N   Q   P   R   V   S   V   G   G   N   D   L   R   N   L   Y   T   L
TTATGGTGGATCCTGATTCACCTAGCCCAAGTAACCCCACTTTTAAGGAGTACCTCCACTGGTTGGTGA
V   M   V   D   P   D   S   P   S   P   S   N   P   T   F   K   E   Y   L   H   W   L   V
CTGATATTCCAGGAACCACTGAAGTCACTTTCGGTAATGAGGTTGTAAATTATGAAAGGCCACGACCCA
T   D   I   P   G   T   T   E   V   T   F   G   N   E   V   V   N   Y   E   R   P   R   P
CTTCGGGGATCCATCGTTTCGTGTTTGTCCTATTTCATCAACAATGTAGACAAAGGGTTTATGCTCCAG
T   S   G   I   H   R   F   V   F   V   L   F   H   Q   Q   C   R   Q   R   V   Y   A   P
GATGGCGACAAAATTTCAACACAAGAGAATTTGCTGAACTCTACAATCTTGGATCACCTGTTGCTGCTG
G   W   R   Q   N   F   N   T   R   E   F   A   E   L   Y   N   L   G   S   P   V   A   A
TCTTCTTCAATTGTCAAAGGGAGAGTGGCTCTGGCGGAAGAACCTTTAGATAATTATTATATTAACTAT
V   F   F   N   C   Q   R   E   S   G   S   G   G   R   T   F   R   *
ATTAAATTAAAATTAAAGTGGAAGCTTAGGGCTGGTTTGGTATCACAATGTATCACCATGAGTCAAGAC
ATTGTAATATCAAACATATGCGTAGTTAAGTTAGTTAGTAATTAAATACTGCAACACCCAAATAAAAGT
TAATTATATTTACATATACACATTATACACTCACACACGCCAAATTAATACACAATTGTAAAAGTAGCA
AATACTAAGTATAGATATGTGTGTCGTTTTATGTTTGTGTATGTATAATGTATCGTATATTATCATCAA
TCAATAAAGAGCTTCCCACTGCCCATTGATATATGTCAGAAAAAAAAAAAAAAAAAAAAAAA
```

Figure S1. The full-length cDNA sequence of *MsFTa* and its encoding protein. The full-length cDNA of *MsFTa* （GenBank: JF681135）with an open reading frame of 531-bp encoding 176 amino acid residues.

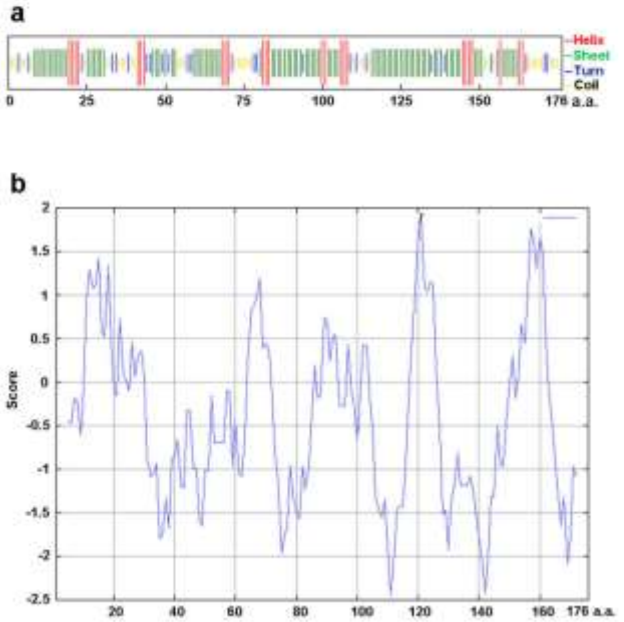

Figure S2. Feature prediction of the deduced MsFTa protein. (**a**). Prediction of the secondary structure of MsFTa using LYON-GERLAND PBIL online software. The color lines denote the secondary structures: helix (red), sheet (green), turn (blue) and coil (yellow). (**b**). Hydropholicity prediction of

MsFTa using ScanProsite online software. Negative values indicate polar amino acids, while positive values for nonpolar amino acids. ScanProsite analysis indicates that MsFTa is a hydrophilic protein.

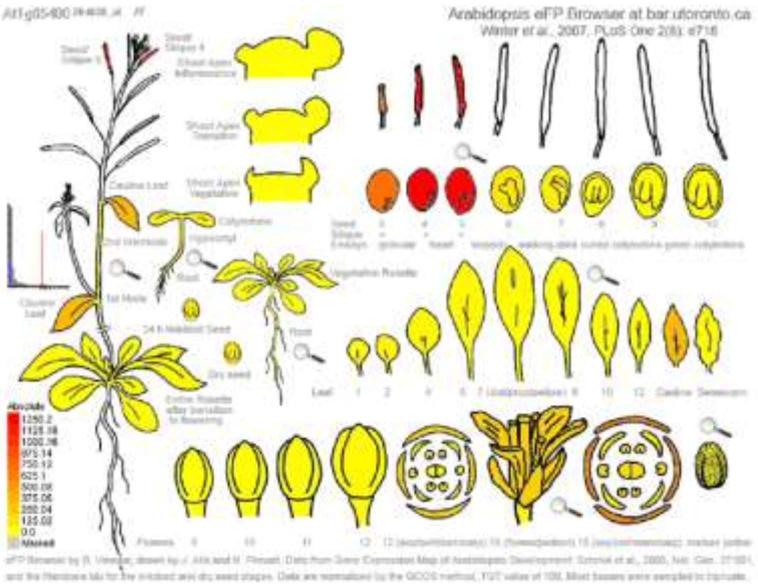

Figure S3. eFP Browser of *AtFT* (*At1g65480*) expression profile during Arabidopsis development.

© 2019 by the authors. Licensee MDPI, Basel, Switzerland. This article is an open access article distributed under the terms and conditions of the Creative Commons Attribution (CC BY) license (http://creativecommons.org/licenses/by/4.0/).
